# Emotional State and Feedback-Related Negativity Induced by Positive, Negative, and Combined Reinforcement

**DOI:** 10.3389/fpsyg.2021.647263

**Published:** 2021-05-10

**Authors:** Shuyuan Xu, Yuyan Sun, Min Huang, Yanhong Huang, Jing Han, Xuemei Tang, Wei Ren

**Affiliations:** ^1^MOE Key Laboratory of Modern Teaching Technology, Shaanxi Normal University, Xi’an, China; ^2^School of Foreign Studies, Anhui Polytechnic University, Wuhu, China

**Keywords:** positive reinforcement, negative reinforcement, dopamine, feedback-related negativity, emotion, electroencephalogram

## Abstract

Reinforcement learning relies on the reward prediction error (RPE) signals conveyed by the midbrain dopamine system. Previous studies showed that dopamine plays an important role in both positive and negative reinforcement. However, whether various reinforcement processes will induce distinct learning signals is still unclear. In a probabilistic learning task, we examined RPE signals in different reinforcement types using an electrophysiology index, namely, the feedback-related negativity (FRN). Ninety-four participants were randomly assigned into four groups: base (no money incentive), positive reinforcement (presentation of money rewards), negative reinforcement (removal of money losses), and combined reinforcement (money rewards and removal of money losses) groups. In addition, in order to evaluate the engagement of emotional activity in the different reinforcement processes, Positive and Negative Affect Schedule-Expanded Form (PANAS-X) scales were applied before and after the experiment to detect the emotional changes. The results showed that there was no difference between groups in the dopamine-related learning bias. However, compared to the other three groups, negative reinforcement elicited smaller FRN (the difference-wave measure) during the learning, stronger positive affect and joviality, and less fatigue after the learning, in which the difference between the negative and positive reinforcement groups was smaller. The results indicated that pure avoidance motivation may induce distinct emotional fluctuations, which influence the feedback processing.

## Introduction

Reinforcement learning is guided by the computation of reward prediction errors [RPEs (Sutton and Barto, 1998)], i.e., the difference between actual outcomes and expectations, which is suggested to be achieved by the midbrain dopamine system (Schultz et al., 1997; Schultz, 1998). Positive RPEs (outcomes are better than expectations) signaled by the phasic dopamine firing facilitate the selection of actions, whereas negative RPEs (outcomes are worse than expectations) by the dopamine dips inhibit it (Frank, 2005). Both the presentation of rewards (positive reinforcement) and the removal of aversive stimuli (negative reinforcement) can elicit positive RPEs. Previous studies showed that increased dopamine activities following unexpected presentation of food (Schultz, 1986) and successful escape from electric shock (Oleson et al., 2012; Wenzel et al., 2015) indicate, respectively, the role of dopamine in both reinforcement types. However, one open issue relates to whether there are differences between the two reinforcement types, considering the reinforcement processes and the underlying mechanisms. It was suggested that the dopamine release is more complicated in negative reinforcement (Wenzel et al., 2015). The investigation of this issue helps to characterize the potential effects of appetitive and aversive stimuli, as well as provide evidence to solve the debate about positive and negative reinforcement (Michale, 1975; Baron and Galizio, 2005, 2006; Chase, 2006; Iwata, 2006; Johnston, 2006; Lattal and Lattal, 2006; Sidman, 2006).

Studies of feedback processing have provided an electrophysiology index for insight into the underlying mechanisms of reinforcement learning, namely, feedback-related negativity [FRN (Miltner et al., 1997)]. This event-related potential (ERP) component occurs at frontal-central sites peaking at around 200–350 ms after performance outcome with a negative deflection for negative compared to positive feedback. The reinforcement learning theory [RL-theory (Holroyd and Coles, 2002)] posits that the FRN reflects a RPE conveyed by the mesencephalic dopamine system to the anterior cingulate cortex (ACC) for filtering the motor controllers. When outcomes are worse than expectations, dopamine dips disinhibit the pyramidal neurons in the ACC, and more negative FRN will be observed. There were studies supporting this theory (Cohen and Ranganath, 2007; Cavanagh et al., 2010). However, evidence has also been found to suggest that the voltage deflection actually derives from a reward positivity induced by positive feedback, but not from negative feedback (Cohen et al., 2007; Holroyd et al., 2008; Holroyd et al., 2011; Sambrook and Goslin, 2016). Walsh and Anderson (2012) reviewed the researches and proposed that although there is a win/loss asymmetry, the FRN reflects a quantitative RPE, in which more negative RPE is associated with more negative FRN and more positive RPE with more positive FRN. A more recent theory, the predicted response-outcome (PRO) model, suggested that the FRN indexes a salience prediction error (SPE, namely, violations of expectancies), regardless of feedback valence (Alexander and Brown, 2011).

The basal ganglia Go/No-Go (BG-GNG) model holds that two parallel pathways inside the basal ganglia gate the action selection with dopamine signals (Frank, 2005; Maia and Frank, 2011). Specifically, positive RPE induces phasic bursts of dopamine neurons to activate the direct pathway, which issues a “Go” signal for the given action, while phasic dips of dopamine after negative feedback activate the indirect pathway, which promotes No-Go learning. A two-phase probabilistic learning paradigm can be used to assess the dopamine-related positive (Go) or negative (No-Go) learning bias (Frank et al., 2004). In the training phase, participants learn through trial and error to choose the more likely correct option over the less one for each stimuli pair. There are three pairs of stimuli with their own corresponding correct probabilities (A-B: 80-20%, C-D: 70-30%, E-F: 60-40%). Then, all pairwise combinations of these six stimuli are presented for choosing without feedback in the test phase, and learning from positive and negative feedback is scored as the accuracy in choosing A and avoiding B, respectively. As predicted by this model, Parkinson’s disease patients have shown a tendency of No-Go learning (Frank et al., 2004), while the dopaminergic medication reversed the bias (Frank et al., 2004; Frank et al., 2007). Consistent with the RL theory, the FRN in the probabilistic learning task was found to be associated with the negative learning tendency (Frank et al., 2005), verifying their common relationship with dopamine.

The FRN responses have been detected in appetitive context with money as positive reinforcers (money delivery and reward omission) and in aversive context with electric shocks or noise bursts as negative reinforcers (aversion omission and aversion delivery) (Crowley et al., 2009; Talmi et al., 2013; Garofalo et al., 2014; Heydari and Holroyd, 2016; Mulligan and Hajcak, 2017; Soder and Potts, 2017). However, the results were not consistent. In a passive task, Talmi et al. (2013) found that unexpected aversion omission induced a more negative voltage deflection than unexpected aversion delivery. However, in a standard pseudo-reinforcement learning task, Heydari and Holroyd (2016) showed that aversion omission induced a reward positivity relative to aversion delivery at a delayed time window and proposed that the effect observed by Talmi et al. (2013) was not related to reward positivity but an early salience effect. For the discrepancy in the nature of money and electric shocks, a direct comparison of appetitive and aversive contexts was seldom done in these studies. Thus, to exclude the salience difference of the reinforcers and compare the reinforcement mechanisms of approach and avoidance motivation (thus, positive and negative reinforcement), we used money reward as a positive reinforcer and money loss as a negative reinforcer in our present study. From the perspective of the context dependence of feedback processing, the FRN responses to the money-related outcomes have also been investigated in the appetitive and aversive contexts (Holroyd et al., 2004; Pfabigan et al., 2015; Zhu et al., 2019). Previous studies showed inconsistent results. Some researches suggested that the unfavorable outcomes elicited a larger FRN than the relatively favorable outcomes in both contexts and that the FRNs were comparable in the two contexts (Holroyd et al., 2004; Angus et al., 2017), indicating similar processing in positive and negative reinforcement. Other studies found that FRN amplitudes were larger for the unfavorable compared to favorable outcomes selectively in the appetitive setting, but not in the aversive context (Santesso et al., 2012; Novak and Foti, 2015), indicating that positive and negative reinforcement may rely on different neural mechanisms. However, all these studies manipulated the conditions in a block- or trial-wise design. It was found that the surrounding context conditions influence the outcome evaluation in the appetitive and aversive settings (Zhu et al., 2019). Thus, separate groups are necessary for investigating the pure effects of the two reinforcement types.

On the other hand, in addition to the dopamine system, the emotional experience may play an important role in the different motivational manipulations. It has long been suggested that positive reinforcement is accompanied by positive emotions, whereas negative reinforcement induces negative emotions (Skinner, 1971; Sidman, 1989; Rakos et al., 2008; Starling et al., 2013). In an applied behavior analysis, positive-reinforcement approaches are regarded as less intrusive and more favorable, while the aversive control including negative reinforcement and punishment is given indictment (Skinner, 1971; Sidman, 1989; Flora, 2004; Schieltz et al., 2019). As Sidman (1989) declared that “People who use punishment become conditioned punishers themselves…. Others will fear, hate, and avoid them. Anyone who uses shock becomes a shock” (p.79). Meanwhile, studies showed that the affective factors modulate feedback processing (Schuermann et al., 2011; Koban and Pourtois, 2014; Paul and Pourtois, 2017). Evidence indicated that negative affect, including anxiety (Gu et al., 2010; Aarts and Pourtois, 2012; Jiang et al., 2018), sadness (Foti and Hajcak, 2010), and depression (Keren et al., 2018), reduces the FRN component, while induced positive mood increases the FRN amplitudes compared to neutral mood (Zhao et al., 2016; Paul and Pourtois, 2017; Bandyopadhyay et al., 2019). The ACC, which is believed as the source of the FRN, has strong connections with extensive structures, including the amygdala, orbitofrontal cortex, and anterior insula, which are all involved in emotional processing (Gaffan and Murray, 1990; Carmichael and Price, 1996; Allman et al., 2006; Koban et al., 2013). It was proposed that the interaction between the ACC and the amygdala is responsible for the automatic emotion tagging of actions (Koban and Pourtois, 2014). Therefore, one possibility is that positive reinforcement may increase the FRN through inducing positive affect and that negative reinforcement may decrease the FRN through eliciting negative affect.

The first goal of the present study was to investigate whether reinforcement type will modulate the FRN and the positive vs. negative learning bias, both of which are suggested to be related to the activity of the midbrain dopamine system. Using money rewards after responses as the positive reinforcement and removal of money deductions as the negative reinforcement, human participants in positive and negative reinforcement groups performed the two-phase probabilistic learning task while electroencephalogram (EEG) was recorded. Besides, we further introduced a base group who performed with no monetary reinforcement as the control condition and a combined reinforcement group who experienced positive and negative reinforcement simultaneously. Second, we aimed to test whether the effect of reinforcement type on the FRN was related to the emotions. Thus, the positive and negative emotions as well as a series of specific affects were assessed using the Positive and Negative Affect Schedule-Expanded Form (PANAS-X) scales (Watson and Clark, 1999). If the emotion system is involved, we predicted that positive reinforcement may elicit positive affect and increase the FRN, whereas negative reinforcement may elicit negative affect and decrease the FRN.

## Materials and Methods

### Participants

Ninety-four healthy undergraduates (35 males, *M*_age_ = 19.38, SD = 1.02) in Shaanxi Normal University, China volunteered to attend the experiment. The criteria for the participant recruitment included the following: (1) right handed, (2) no self-reported history of major brain trauma and mental disorders, (3) normal or rectified-to-normal vision, and (4) native speakers of Chinese with no experience in learning Japanese. Participants were randomly assigned to one of the four groups: base group (eight males and 17 females), positive reinforcement group (nine males and 13 females), negative reinforcement group (eight males and 14 females), and combined reinforcement group (10 males and 15 females). At the time of recruitment, participants were informed that the remuneration ranged from 30 to 70 RMB. Unknown to participants, the actual remuneration ranged from 45 to 70 RMB. If participants did not reach the learning criteria and had less than 45 RMB in their accounts, they received the minimum payment (one participant in the negative reinforcement group). And, if participants reached the learning criteria and had more than 70 RMB in their accounts, they were paid the maximum as the advertisement claimed (10 participants in the positive reinforcement group). Participants were paid when they left the laboratory. All participants provided written, informed consent, and the research ethics committee of Shaanxi Normal University approved the study.

### Procedure

The experiment procedure was as follows: After the preparatory work, a pretest of emotions using the PANAS-X was conducted. Then, four separate minutes of baseline EEG were recorded; 2 min with eyes open and 2 min with eyes closed, in a counterbalanced order (data is not reported here). Subsequently, the participants performed the probabilistic learning task, during which EEG was recorded. Finally, an identical posttest of emotions using the PANAS-X was completed.

#### Probabilistic Learning Task

**FIGURE 1:**
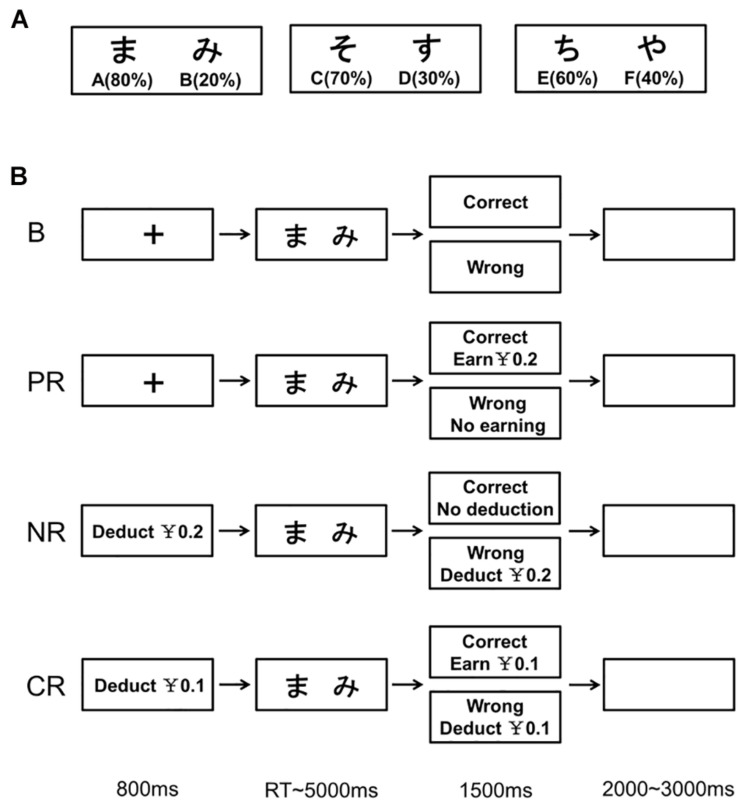
Overview of the training phase in the probabilistic learning task. **(A)** Three pairs of stimuli (Japanese characters) used in the task. The figures below the characters show corresponding probabilities to be correct within their own pairs. **(B)** Manipulation of monetary incentives on the training trials in the four reinforcement types. B, base group; PR, positive reinforcement; NR, negative reinforcement; CR, combined reinforcement.

The probabilistic learning task consisted of two phases: the training phase and the test phase (Frank et al., 2005; Schmid et al., 2017). In the training phase, pairs of stimuli (Japanese characters) were presented (Figure 1A). Participants were asked to choose one stimulus on each trial and would receive “correct” or “wrong” feedback after choosing. However, the feedback was probabilistic, and it varied between three object pairs: The correct probabilities for A-B, C-D, and E-F were 80-20%, 70-30%, and 60-40%, respectively (Figure 1A). That is, for example, A was “correct” in the 80% of AB trials and B was “correct” in the remaining 20%. Through trial and error, participants learned to choose A over B, C over D, and E over F. To achieve this, participants may learn through choosing A (learning from positive feedback) or avoiding B (learning from negative feedback). The training phase included up to six blocks of 60 trials each. Participants would proceed to the test phase if the learning criteria were met or the number of completed blocks reached six. Following Frank et al. (2005), the criteria to enter the test phase were 65% correct rate for AB trials, 60% for CD trials, and 55% for EF trials. For those automatically entering the test phase, the number of learning blocks was marked as “7” (Schmid et al., 2017).

The training trial for the base group started with a fixation cross of 800 ms. Then, the stimuli pair was presented with a maximum of 5,000 ms. Once the participants pressed the button (“F” key for the left stimulus with their left index finger or “J” key for the right stimulus with their right index finger) to choose one of them or the time was up, feedback (“correct” or “wrong”) was presented for 1,500 ms. In the case of no response, the feedback was “wrong.” The inter-trial intervals jittered between 2,000 and 3,000 ms. The sequence of the three stimuli pairs in each block was random. The left–right placement order of stimuli in each pair was counterbalanced across trials, and the correspondence between stimuli and the correct probabilities was counterbalanced across participants. During the breaks between blocks, the resting time was at will. For the task, all participants of the four groups were instructed to learn through trials which stimuli were more likely to be correct than the others and choose those associated with a higher correct probability in each pair.

The trial procedure was the same across groups, but the money-related settings varied (Figure 1B). At the beginning of the training phase, participants in the base group were instructed that their remuneration was 50 RMB, which was independent of their performances, whereas the other three groups were told that they initially had 50 RMB in their accounts and would earn (for the positive reinforcement group) or lose money (for the negative reinforcement group and both for the combined reinforcement group) according to their performances. For the positive reinforcement group, they could earn 0.2 RMB on each “correct” trial but no money reward on the “wrong” trials. For the negative reinforcement group, the instruction was that “At the start of each trial, 0.2 RMB will be deducted from your account. On trials with positive feedback, the deducted money will be returned.” For the combined reinforcement group, participants were instructed that “At the start of each trial, 0.1 RMB will be deducted from your account. On trials with positive feedback, not only the deducted money will be returned, but also you will earn another 0.1 RMB.” Besides, for the probabilistic nature of the task, participants were told that the reward would take effect (for the positive reinforcement group) and all the deducted money would be returned (for the negative reinforcement group and both for the combined reinforcement group) only if the learning criteria were reached and that it was the reverse if the criteria were not reached. At the end of each training block, during the break time, the account balance and whether they had reached the criterion were presented (for the base group, only the latter).

In the test phase, the stimuli pairs became all the 15 pairwise combinations of the six stimuli, each of which was presented six times in only one block (90 trials in total). No feedback was provided, and no reaction time limitation was set. Beyond these, the test trials were the same as the training phase of the base group. Participants were instructed again to choose the stimuli that they believed had a higher probability to be correct in novel pairs. In case they were unsure, they were asked to respond based on intuition.

To assess the reinforcing effect, the learning speed and effect were characterized by the number of blocks completed in the training phase and the overall accuracy in the test phase, which was the ratio of correct responses on all test trials, respectively. The correct response on each trial was choosing the stimulus with a higher correct probability over the lower, which was learned in training. Then, the learning bias was investigated. Learning from positive feedback was measured as the accuracy of choosing A over all the other stimuli in the test phase, while learning from negative feedback was identified as the accuracy of avoiding B. The learning bias was calculated as positive learning scores minus negative learning scores, with a positive value indicating a positive learning bias and a negative value suggesting a negative bias.

### Emotion Measurement

The Positive and Negative Affect Schedule (PANAS) includes two high-level general-dimension scales, the 10-item Positive Affect (PA) and Negative Affect (NA) scales, to assess the valence of mood (Watson and Tellegen, 1985; Watson et al., 1988). The PANAS-X expands the basic version, adding 11 lower-level specific affect scales (Watson and Clark, 1999) to measure the content of mood. In total, it includes 60 mood descriptors, such as happy and sad. On each item, subjects were asked to evaluate the extent to which they had these feelings during the specific time frame on a five-point Likert scale (1 = very slightly or not at all and 5 = extremely). Then, accumulative scores on the included items of each scale indicate how much they felt this emotion.

In the present study, to detect the variation in emotion induced by different reinforcement types, participants were asked to report their momentary feelings pre- and post-task using the Chinese revision of PANAS-X scales. Zhang et al. (2004) found that, although the two-factor dimensionality was valid across culture, the “alert” item in PA scales has cross-factor loading, indicating the cultural bias. The PA and NA scales used here were the Chinese reversion of PANAS by Qiu et al. (2008), which includes nine items in each subscale. On the other hand, Guo and Gan (2010) revised the specific affect scales in PANAS-X. Because of the low power of two factors in the exploratory factor analysis, the 11 scales are reduced to nine, and they are hostility, guilty, sadness, joviality, self-assurance, shyness, fatigue, serenity, and surprise. Furthermore, the original 55 descriptors in the specific affect scales are reduced to 40 items because of low loading. Finally, the Chinese revision scales consist of 51 items, with two general dimension scales and nine specific affect scales.

The Cronbach’s alphas of the subscales were as follows: positive affect (0.914), negative affect (0.830), hostility (0.863), guilty (0.867), sadness (0.806), joviality (0.940), self-assurance (0.876), shyness (0.817), fatigue (0.903), serenity (0.309), and surprise (0.836). Except for the serenity subscale, all the remaining subscales had a good reliability. The emotion change was measured as the post-test scores minus the pre-test scores.

### EEG Recording and Preprocessing

The EEG activity was recorded from 64 scalp locations, using Ag/AgCl electrodes mounted in an elastic cap (Quik-Cap, Neuroscan, El Paso, TX) with a standard 10–20 layout. The electrodes were referenced to the left mastoid, and all the impedances were maintained below 5 kΩ. The vertical and horizontal electroocolugrams were recorded from electrodes placed supraorbitally and infraorbitally at the left eye and on the outer canthus of each eye, respectively. Signal was amplified using Neuroscan Synamps2 (El Paso, TX) with AC coupling, digitized at 1,000 Hz, and monitored on the Curry 7 software (Neuroscan, El Paso, TX). Offline, the data processing was completed on the MATLAB through EEGLAB 14.1.1 and ERPLAB 7.0.0 toolbox. The EEG data were re-referenced to average mastoids mathematically. The vertical and horizontal ocular artifacts were identified through an independent component analysis (ICA) and removed from the continuous EEG data.

For ERPs, data was filtered through a 0.1–30-Hz band-pass Butterworth filter. Then, 1,000-ms epochs were extracted around the feedback onset from -200 to 800 ms and baseline corrected over a window of −200–0 ms prior to the feedback onset. On average, 2.7% of the epochs were rejected using simple voltage thresholds of ± 75 μV. ERPs were produced by averaging EEG epochs across trials in each condition at each electrode for each participant. To have a comprehensive investigation of the FRN, we used three scoring methods to measure it at FCz, Cz, and Pz. The peak-to-peak measure defined the FRN as the voltage difference between the negative peak (within 230–400 ms after feedback onset) and the preceding positive peak (within 180 ms to the negative peak). When no negative peak was found, the amplitude was set to 0 μV. The mean amplitude measure averaged the voltage value within the time window of 290–350 ms after feedback onset. In addition, difference waves were generated by subtracting the correct ERPs from the wrong ERPs. The negative peak of the difference waves within 200–400 ms after feedback onset was detected for the difference wave measure. On the other hand, the FRN is susceptible to component overlap with the P300. To assess this issue, we also investigated the P300, which was measured as the most positive peak in the time window of 200–600 ms following feedback onset.

### Statistics Analysis

For behavioral performance, because the completed number of blocks was discrete, a nonparametric statistical method, Kruskal–Wallis test, was conducted to test the effect of the reinforcement type on learning speed. The overall accuracy and the learning bias were analyzed using a four-level (the four reinforcement types) one-way ANOVA. Positive and negative learning scores were entered into a 4 (the four reinforcement types) × 2 (learning tendency: positive learning and negative learning) two-way ANOVA.

To assess whether the emotions were modulated by the reinforcement type, we first conducted a MANOVA on the emotion change scores from the 10 subscales (the serenity scores were excluded from analysis for the low reliability). In addition, ANCOVAs with the pre-test scores as the covariates were used to test the post-test scores to confirm the results for each subscale.

As participants learned the stimuli A, C, and E were associated with higher reward probabilities, choosing these stimuli would lead to expected positive feedback or unexpected negative feedback and choosing B, D, and F would lead to unexpected positive feedback or expected negative feedback. Because the FRN is believed to index RPEs, expectation should modulate it. Thus, the peak-to-peak and mean amplitude measures of the FRN were analyzed using 4 (the four reinforcement types) × 2 (feedback valence: correct and wrong) × 2 (expectation: expected and unexpected) × 3 (location: FCz, Cz, and Pz) four-way ANOVAs. Then, the difference wave measure of the FRN was analyzed using a 4 (the four reinforcement types) × 2 (expectation: expected and unexpected) × 3 (location: FCz, Cz, and Pz) three-way ANOVA. The P300 was assessed using the four-way ANOVA as well. Four participants (one, two, and one in the base group, positive reinforcement group, and combined reinforcement group, respectively) were excluded from the EEG analyses for less than three trials in the unexpected correct condition. For those who advanced to the test phase directly, we found that the test accuracy was above chance as well [*t*(9) = 5.82, *p* < 0.001), indicating learning in the training phase. Thus, we included these participants in the EEG analyses. The Greenhouse–Geisser correction was used to adjust the degrees of freedom.

## Results

### Behavioral Results

For all participants, the mean value of the number of learning blocks was 3.59 (SD = 1.80). Totally, 10 participants (*n*_Base_ = 5, *n*_Positive_ = 3, *n*_Negative_ = 1, *n*_Combined_ = 1) did not reach the criteria and advanced to the test phase directly. One-sample *t* test revealed that the test accuracy (*M* = 0.76, SD = 0.13) was above chance [*t*(93) = 20.02, *p* < 0.001], indicating that the task was successful in inducing learning.

**FIGURE 2:**
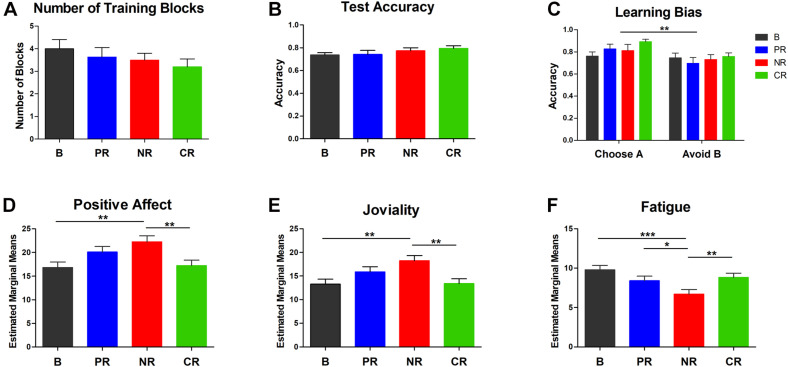
The behavior performance and the estimated marginal means of three affects at the end of the experiment in ANCOVAs with the pre-test scores as the covariates. **(A)** The number of blocks completed in the training phase. **(B)** The overall accuracy in the test phase. **(C)** Learning from positive (the accuracy of choosing A) and negative feedback (the accuracy of avoiding B). An overall positive learning bias was found. For the above three indexes, no difference was found in the four reinforcement types. **(D-F)** Participants in the negative reinforcement group reported higher positive affect, higher joviality, and lower fatigue after the experiment. Error bars in all panels represent SEM. Asterisks represent differences at a significance level of *0.05; **0.01; ***0.001. B, base group; PR, positive reinforcement; NR, negative reinforcement; CR, combined reinforcement.

Figure 2A depicts the number of learning blocks in the four reinforcement types (*M*_Base_ = 4.00, SD = 2.00, *M*_Positive_ = 3.64, SD = 1.92, *M*_Negative_ = 3.50, SD = 1.37, *M*_Combined_ = 3.20, SD = 1.72), and Figure 2B shows the overall test accuracy (*M*_Base_ = 0.74, SD = 0.10; *M*_Positive_ = 0.74, SD = 0.16; *M*_Negative_ = 0.77, SD = 0.12; *M*_Combined_ = 0.79, SD = 0.11). Less learning blocks needed and higher test accuracy mean more effective reinforcement. Although the descriptive data showed a gradient of reinforcing effect with combined reinforcement, negative reinforcement, positive reinforcement, and no money reinforcement in descending order, there was no statistically significant difference in the block number [χ^2^ (3) = 2.00, *p* = 0.573] and the overall accuracy [*F*(3,90) = 1.15, *p* = 0.335, η_p_^2^ = 0.037]. Chi-square analysis also showed that there was no difference between groups in the percentage of participants who did not meet the criteria [χ^2^ (3) = 5.09, *p* = 0.166]. Thus, the four reinforcement types had comparable reinforcement effect in this probabilistic learning task.

For learning from positive and negative feedback, the two-way ANOVA showed a main effect of learning tendency [*F*(1,90) = 9.50, *p* = 0.003, η_p_^2^ = 0.095], but no reinforcement type effect [*F*(3,90) = 1.13, *p* = 0.342, η_p_^2^ = 0.036] and no interaction effect [*F*(3,90) = 0.97, *p* = 0.410, η_p_^2^ = 0.031]. The effect of learning tendency indicated that participants learned better from positive feedback (*M* = 0.82, SD = 0.20) than from negative feedback (*M* = 0.73, SD = 0.21, Figure 2C). A one-way ANOVA on the learning bias scores confirmed that there was no significant difference between groups [*F*(3,90) = 0.97, *p* = 0.411, η_p_^2^ = 0.031]. Thus, positive or negative money reinforcement did not influence the learning bias in the present study.

### Emotions

The MANOVA showed that the emotion change before and after the experiment was modulated by reinforcement type [*F*(30,249) = 1.78, *p* = 0.010, η_p_^2^ = 0.176], and the variations between groups resulted from positive affect [*F*(3,90) = 7.09, *p* < 0.001, η_p_^2^ = 0.191], joviality [*F*(3,90) = 7.70, *p* < 0.001, η_p_^2^ = 0.204], and fatigue feeling [*F*(3,90) = 3.90, *p* = 0.011, η_p_^2^ = 0.115], but not from negative affect, hostility, guilty, sadness, self-assurance, shyness, and surprise (*p* > 0.176). Consistent with this, ANCOVAs also revealed that positive affect [*F*(3,89) = 4.27, *p* = 0.007, η_p_^2^ = 0.126, Figure 2D], joviality [*F*(3,89) = 4.69, *p* = 0.004, η_p_^2^ = 0.137, Figure 2E], and fatigue feeling [*F*(3,89) = 5.31, *p* = 0.002, η_p_^2^ = 0.152, Figure 2F] were modulated by reinforcement type. However, contrary to our hypothesis, participants in the negative reinforcement group reported higher levels of positive affect and joviality than the base group (*p*_positive–affect_ = 0.003 and *p*_joviality_ = 0.002) and the combined reinforcement group (*p*_positive–affect_ = 0.004 and *p*_joviality_ = 0.002) at the end of the experiment. A trend for a higher increase of joviality in the negative compared to the positive reinforcement group was also found in the *post hoc* multiple comparisons of MANOVAs (*p* = 0.089). Moreover, fatigue feeling after the task was lower for the negative reinforcement group than the other three groups (*p*_Base–Negative_ < 0.001, *p*_Positive–Negative_ = 0.041, *p*_Combined–Negative_ = 0.009). To conclude, negative reinforcement increased positive affect and joviality and decreased fatigue feeling after the experiment compared with no-money-incentive and combined reinforcement types, and negative reinforcement decreased fatigue feeling compared with positive reinforcement.

### Feedback-Related Negativity

**FIGURE 3:**
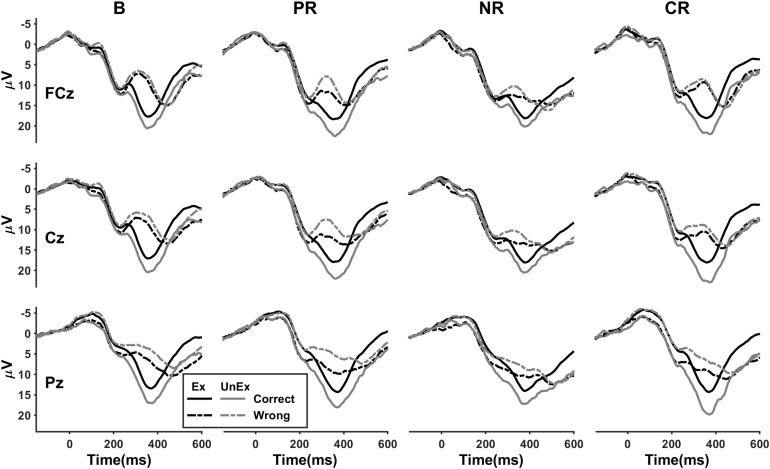
The condition-specific event-related potential (ERP) waveforms for four reinforcement types at FCz, Cz, and Pz. B, base group; PR, positive reinforcement; NR, negative reinforcement; CR, combined reinforcement; Ex, expected; UnEx, unexpected.

**FIGURE 4:**
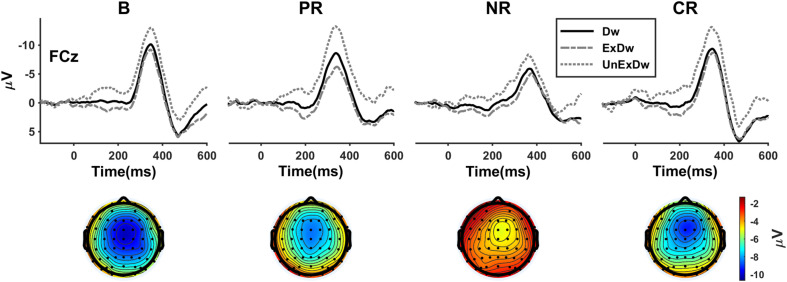
The difference waves for four reinforcement types at FCz and the corresponding topographies showing the mean amplitudes of the overall difference waves (black solid line) in the time window of 340–350 ms after feedback onset. B, base group; PR, positive reinforcement; NR, negative reinforcement; CR, combined reinforcement; Dw, difference wave; ExDw, expected difference wave; UnExDw, unexpected difference wave.

In line with previous researches, a “wrong” feedback elicited a more negative deviation relative to “correct” at roughly 350 ms after feedback (Figure 3). The FRN had a frontal-central topography and was maximal at FCz (Figure 4). For the peak-to-peak measure of the FRN, the four-way ANOVA indicated a significant main effect of feedback valence [*F*(1,86) = 117.93, *p* < 0.001, η_p_^2^ = 0.578] in which the amplitudes were more negative following a “wrong” feedback than following a “correct” feedback. A significant main effect of expectation [*F*(1,86) = 31.79, *p* < 0.001, η_p_^2^ = 0.270] showed that the unexpected condition induced larger FRN than the expected. The main effect of location was also significant [*F*(1.1,95.3) = 77.67, *p* < 0.001, η_p_^2^ = 0.475], in which the FRN was larger at FCz than at Cz (*p* < 0.001) or Pz (*p* < 0.001) and larger at Cz than at Pz (*p* < 0.001). The main effect of reinforcement type was not significant [*F*(3,86) = 1.41, *p* = 0.246, η_p_^2^ = 0.047]. However, an expectation × reinforcement type interaction [*F*(3,86) = 3.03, *p* = 0.034, η_p_^2^ = 0.096] indicated that the amplitudes were larger for the base group than for the positive (*p* = 0.004) and combined reinforcement group (*p* = 0.007) in the expected condition [*F*(3,86) = 3.75, *p* = 0.014, η_p_^2^ = 0.116], but not in the unexpected condition [*F*(3,86) = 0.37, *p* = 0.776, η_p_^2^ = 0.013]. The difference between the base group and the negative reinforcement group in the expected condition was marginally significant (*p* = 0.052).

For the mean amplitude measure of the FRN (Figure 3), the four-way ANOVA also revealed a significant main effect of valence [*F*(1,86) = 208.07, *p* < 0.001, η_p_^2^ = 0.708]. The main effect of location [*F*(1.2,102.2) = 128.50, *p* < 0.001, η_p_^2^ = 0.599] indicated that the averaged voltage value was larger at FCz (*p* < 0.001) or Cz (*p* < 0.001) than at Pz. The main effect of expectation was not significant [*F*(1,86) = 3.30, *p* = 0.073, η_p_^2^ = 0.037]. However, a highly significant interaction between valence and expectation was found [*F*(1,86) = 82.18, *p* < 0.001, η_p_^2^ = 0.489]. The unexpected wrong condition induced more negative amplitudes than the expected wrong [*F*(1,86) = 63.83, *p* < 0.001, η_p_^2^ = 0.426], whereas the unexpected correct condition was associated with more positive amplitudes than the expected correct [*F*(1,86) = 52.31, *p* < 0.001, η_p_^2^ = 0.378]. The main effect of reinforcement type was not significant [*F*(3,86) = 0.95, *p* = 0.422, η_p_^2^ = 0.032], but a valence × reinforcement type interaction was found [*F*(3,86) = 5.57, *p* = 0.002, η_p_^2^ = 0.163]. Following “wrong” feedback, the effect of reinforcement type reached a critical level [*F*(3,86) = 2.59, *p* = 0.058, η_p_^2^ = 0.083], but not following “correct” feedback [*F*(3,86) = 0.86, *p* = 0.467, η_p_^2^ = 0.029]. Pairwise comparisons indicated that the amplitudes were more negative for the base group than for the negative reinforcement group (*p* = 0.008).

We finally analyzed the difference wave measure of the FRN (Figure 4). The three-way ANOVA showed a significant main effect of expectation [*F*(1,86) = 104.97, *p* < 0.001, η_p_^2^ = 0.550]. The unexpected difference waves were more negative than the expected. The main effect of location [*F*(1.2,99.8) = 31.88, *p* < 0.001, η_p_^2^ = 0.270] revealed that the FRN was larger at FCz than at Cz (*p* = 0.036) or Pz (*p* < 0.001) and larger at Cz than at Pz (*p* < 0.001). The expectation × location interaction effect [*F*(1.2,103.3) = 10.55, *p* = 0.001, η_p_^2^ = 0.109] showed that the location effect remained at the unexpected condition [*F*(2,85) = 30.04, *p* < 0.001, η_p_^2^ = 0.414], but at the expected condition [*F*(2,85) = 9.40, *p* < 0.001, η_p_^2^ = 0.181], only the difference between Cz and Pz was significant (*p* = 0.004). The main effect of reinforcement type was significant [*F*(3,86) = 4.04, *p* = 0.010, η_p_^2^ = 0.123], in which the FRN response of the negative reinforcement group was significantly smaller than that of the base group (*p* = 0.005) and combined reinforcement group (*p* = 0.002). Although the positive reinforcement group appeared to show larger FRN than the negative, the difference did not reach statistical significance (*p* = 0.074). With a more lax standard, separate *t* tests on the difference waves between the positive and negative reinforcement groups at FCz were significant for the overall [*t* (40) = −2.08, *p* = 0.044] and the unexpected [*t* (40) = −2.47, *p* = 0.018], but not for the expected [*t* (40) = −0.72, *p* = 0.478]. There was also a significant interaction between reinforcement type and location [*F*(3.5,99.8) = 2.89, *p* = 0.032, η_p_^2^ = 0.091], which revealed that the location effect was significant for the base group [*F*(2,85) = 7.49, *p* = 0.001, η_p_^2^ = 0.150] and combined reinforcement group [*F*(2,85) = 17.66, *p* < 0.001, η_p_^2^ = 0.294], but not for positive [*F*(2,85) = 2.74, *p* = 0.070, η_p_^2^ = 0.061] and negative reinforcement [*F*(2,85) = 1.31, *p* = 0.276, η_p_^2^ = 0.030].

### P300

The four-way ANOVA of P300 showed a reliable main effect of valence [*F*(1,86) = 106.08, *p* < 0.001, η_p_^2^ = 0.552], indicating the P300 was larger for “correct” feedback than for “wrong” feedback. Although the main effect of expectation was significant [*F*(1,86) = 60.88, *p* < 0.001, η_p_^2^ = 0.414], it also had an interaction with valence [*F*(1,86) = 81.47, *p* < 0.001, η_p_^2^ = 0.486]. The unexpected correct condition was associated with larger P300 as compared to the expected correct [*F*(1,86) = 116.8, *p* < 0.001, η_p_^2^ = 0.576], whereas the P300 of the unexpected wrong condition was smaller than the expected wrong [*F*(1,86) = 11.55, *p* < 0.001, η_p_^2^ = 0.118]. In addition, a significant main effect of location [*F*(1.2,104.7) = 196.26, *p* < 0.001, η_p_^2^ = 0.695] revealed that the P300 was larger at FCz than at Cz (*p* < 0.001) or Pz (*p* < 0.001) and larger at Cz than at Pz (*p* < 0.001). The results suggested that the P300 in the present study showed a frontal-central topography, and the modulation effects of valence and expectation were similar on the P300 and on the mean amplitude measure of the FRN. Thus, the FRN may have a greater influence on the P300, but not the reverse, and there were no significant effects related to reinforcement type on the P300 (*p* > 0.205).

### Correlations

In the above analyses, the modulation effects of reinforcement type on the emotions and the FRN were similar. So, Pearson correlation analyses were conducted between the emotion change scores (changes of positive affect, joviality, and fatigue) and the difference wave measure (the overall, expected, and unexpected) of the FRN at FCz. For the overall difference waves (wrong-correct), smaller amplitudes were associated with more increase of joviality after finishing the experiment (*r* = 0.215, *p* = 0.042). There was no significant correlation between the expected difference waves and the emotion changes (*p* > 0.255). However, for the unexpected difference waves, smaller amplitudes were associated with more increase of joviality (*r* = 0.230, *p* = 0.029) and less increase of fatigue feeling (*r* = −0.222, *p* = 0.036). Nevertheless, the correlations were no longer significant after Bonferroni correction. There was no correlation between the emotion change scores and the test accuracy or the P300 (*p* > 0.143).

## Discussion

In the present study, our aim was to identify the distinction in the neural mechanisms underlying positive and negative reinforcement. To these ends, we compared the effects of four money-related reinforcement types, including reinforcement with no money incentive, positive, negative, and combined reinforcement, on the positive versus negative learning bias, the self-reported emotion changes, and the FRN in a probabilistic learning paradigm. The results showed that there was no difference in the learning bias among the four groups, while the induced emotions varied. However, contrary to our prediction, higher positive affect and joviality and lower fatigue were reported after the experiment in negative reinforcement compared to the no-money-incentive and combined reinforcement types and lower fatigue in negative reinforcement compared to positive reinforcement. We used three methods to measure the FRN. The peak-to-peak amplitudes were larger for the no-money-incentive group than for the other three groups, specifically in the expected condition. The mean amplitudes following “wrong” feedback were larger for the no-money-incentive group than for the negative group. Finally, the difference waves were smaller for the negative reinforcement group than for the other three groups, in which the difference between the positive and negative reinforcement groups was smaller. Though relatively weak, the correlation analyses revealed that smaller unexpected difference waves were associated with higher increase of joviality and decrease of fatigue after the experiment.

### The Reinforcement Types and the Dopamine-Related Responses

Our finding that the learning bias was similar in the four reinforcement types suggested that only valence information was utilized for learning in our present task. Positive reinforcement did not bias the learning to increase the probability of choosing the more likely rewarded stimuli, and negative reinforcement did not potentiate the avoidance tendency as well. Rather, a positive learning bias was found for all groups. According to the BG-GNG model (Frank, 2005), phasic dopamine release will potentiate the activation of the direct pathway and inhibit the indirect pathway to facilitate the selection of actions, while the phasic dips of dopamine in the striatum will suppress the direct pathway and activate the indirect pathway to issue a No-Go signal. Using optogenetics, Kravitz et al. (2012) proved that mice repeated the behavior that could specifically stimulate the striatal direct neurons, while that activating the striatal indirect cells was inhibited. In congruence with this model, the dopamine level has been associated with the bias to learning from positive vs. negative feedback (Frank et al., 2004; Frank et al., 2007). Thus, the positive learning bias in our present study may reflect a tonic dopamine level. However, the dopamine-related difference between positive and negative reinforcement was not present at the behaviors.

Our result that the FRN response was more profound following “wrong” than following “correct” feedback was in line with previous researches (Frank et al., 2005; Yeung et al., 2005; Cohen et al., 2007; Holroyd and Krigolson, 2007; Mas-Herrero and Marco-Pallarés, 2014). Using the FRN as the electrophysiology index, we sought to investigate RPE signals in positive and negative reinforcement. However, previous studies were inconsistent with the functional significance of the FRN, namely, whether the FRN reflects positive RPEs (Sambrook and Goslin, 2016), negative RPEs (Holroyd and Coles, 2002), quantitative RPEs (Walsh and Anderson, 2012), or SPEs (Alexander and Brown, 2011). Thus, we included the factor expectation in the analyses. We found that the peak-to-peak amplitudes were more negative in the unexpected condition than in the expected condition for both positive and negative outcomes, indicating it is a SPE signal, whereas the valence × expectation interaction effect proved that the mean amplitudes index quantitative RPEs. The results suggested that different measures of the FRN showed distinct representations, which may explain the inconsistence in previous researches. As the peak-to-peak measure of the FRN showed characteristics of SPEs, the larger amplitudes in the expected condition for the no-money-incentive group than for the other three groups suggested that the expected outcomes were evaluated as less salient under money-manipulated reinforcement. As the mean measure of the FRN was sensitive to quantitative RPEs, the result that the amplitudes following “wrong” feedback were larger for the no-money-incentive group than for the negative group indicated that the negative RPEs were decreased in negative reinforcement.

### The Reinforcement Types and the Emotions

For the aversive nature of negative reinforcers, negative reinforcement was considered as unpleasant, intrusive, and having negative side effects (Skinner, 1971; Sidman, 1989; Flora, 2004; Starling et al., 2013). Our finding that participants in the negative reinforcement group reported higher positive affect and joviality after finishing the task than the no-money-incentive and combined reinforcement groups, and lower fatigue feeling than the other three groups, appeared to contradict to this notion. Unlike animals suffering electric shocks in negative reinforcement, the experience of which could not be erased once the shocks had been administrated, our rules permitted participants to recover all the potential losses if they reached the criteria. According to the prospect theory in the domain of economics, people tend to be risk loving in decision making involving losses and be risk aversive in decision making involving gains (Tversky and Kahneman, 1992). Losses are weighted twice, psychologically, as gains, the phenomenon of which is referred as loss aversion (Kahneman and Tversky, 1984). It implies that losing a certain amount of money will decrease more satisfaction than gaining the same amount of money, and if the losses can be avoided, the satisfaction will be more than gaining money. The results in the present study were consistent with this theory. The success of loss avoidance made participants in the negative reinforcement group feeling better not only than the no-money-incentive group but also than the positive and combined reinforcement. So, the results suggested that when the loss can be completely recovered, negative reinforcement would bring more positive feelings after training. However, our investigations did not allow the propagation of this conclusion to other time frames.

Our daily experience always encompasses such scene: we do not like aversive events; however, once they are terminated, it makes us feel a sense of relief or even joviality. The opponent-process theory of Solomon (1980) depicted the picture of emotion contrast in detail. The affective reaction to the presentation and removal of the unconditioned stimuli goes through the contrast process. Once the unconditioned stimuli (pleasant or aversive) are presented, the corresponding emotion (positive or negative) is evoked, then increased and finally declines to a steady level after a peak; then, as the stimuli are removed subsequently, the emotion that contrasts to the previous stage emerges; finally, it goes back to the original baseline. Furthermore, the theory also revealed that after many times of repeat, the reaction to the unconditioned stimuli will diminish while the contrast reaction after the removal of the unconditioned stimuli will be prolonged and magnified. In the current study, participants in negative reinforcement might go through the contrast period. Thus, we speculated that, during the task, the negative reinforcement group went through more negative emotion, and when the task was over, they experienced more relief and joviality. This speculation can be supported by the examination of the FRN and its relation to emotion.

Although the inverse problem has no unique solution, both dipole and distributed source modeling studies have suggested that the main neural source that generates the FRN is the ACC (Ruchsow et al., 2002; Doñamayor et al., 2011). Besides action monitoring and cognitive control, the ACC is an interactive hub that has also been involved in emotional processing (Etkin et al., 2011; Shackman et al., 2011). This was supported by its broad connections with many emotion-related areas, including the amygdala, orbitofrontal cortex, and anterior insula (Gaffan and Murray, 1990; Carmichael and Price, 1996; Allman et al., 2006; Koban et al., 2013). Koban and Pourtois (2014) proposed that the interaction between the ACC and amygdala is responsible for the automatic affective tagging of actions. Consistent with this, previous researches confirmed the emotion–cognitive interaction on the FRN (Koban and Pourtois, 2014). A smaller amplitude of the FRN (difference wave measure) was associated with less interest rating in a no-response task compared to a response task (Yeung et al., 2005). Studies on the anxious participants showed that high-trait anxiety elicited a blunted FRN compared to low-trait anxiety (Gu et al., 2010; Aarts and Pourtois, 2012; Jiang et al., 2018), whereas positive mood induced by guided imagery increased the FRN amplitude (Paul and Pourtois, 2017). Therefore, our finding that the difference wave amplitudes of the FRN were smaller in the negative reinforcement group may result from the experienced negative emotion. Though not very strong, the correlations between the difference waves and the emotion changes were found. A complete picture may be that, compared to the other three reinforcement types, negative reinforcement elicited more negative affect in the training process, which had an influence on the feedback processing in the ACC, but more positive affect after the experiment.

In previous FRN researches using shocks or noise bursts as negative reinforcers, some reported that aversion delivery induced a more positive voltage deflection than aversion omission, especially in passive tasks (Talmi et al., 2013; Soder and Potts, 2017). Our finding that emotions may be involved in the modulation of the FRN following a “wrong” feedback by negative reinforcement provided insight to previous results. The more positive FRN following aversion delivery may partially be due to the negative emotions induced by the incoming shocks or noise bursts. Thus, the emotion and motivation process may be more complicated in negative reinforcement.

### Distinction Among Reinforcement Types

Based on the present results, no difference was detected among different reinforcement types concerning behavioral performance. It has been suggested that positive reinforcement and negative reinforcement are mathematically equivalent under variable interval schedules. In other words, the optimal behaviors are identical in the two cases (Mallpress et al., 2012). Perone (2003) also proposed that positive reinforcement and negative reinforcement were comparable in strengthening behavior. Our results agree with this proposal. However, our findings indicated that negative reinforcement induced different emotional reactions relative to positive and combined reinforcement. The combination of positive and negative reinforcement did not act in a simple additive way. It suggested that motivation-related contexts can be divided into two kinds: (1) situations with the chance to obtain reward (no matter the negative reinforcers existed or not) and (2) just act to escape from losing or suffering. Compared with situations with the chance to obtain reward, pure motivation to avoid bad things had a different effect on emotional experience and the emotion-modulated EEG signals. Thus, the difference between positive and negative reinforcement lies in the emotional influences that lurk beneath the surface to have a potential impact on individuals. Chronic and long-term negative reinforcement may have a profound effect on emotion and personality development. An in-depth understanding of the different effects of positive reinforcement and negative reinforcement needs more efforts in the future.

There is a debate on positive and negative reinforcement. Michale (1975) argued that the distinction of positive reinforcement and negative reinforcement and that of positive punishment and negative punishment should be abolished. In sum, he had two considerations: confusion in terminology and difficulty in distinguishing between the concepts according to presenting and removing in complex human situations. Baron and Galizio (2005) raised anew the issues and concluded that no new evidence supported the distinction by reviewing research progress during the past 30 years. For the lack of evidence, the discussion of this issue in 2006 did not conclude (Baron and Galizio, 2006; Chase, 2006; Iwata, 2006; Johnston, 2006; Lattal and Lattal, 2006; Marr, 2006; Sidman, 2006). Our results that the emotion involved in the pure negative reinforcement was distinct, which had an influence on feedback processing, supported that these concepts should be retained. Based on a free operant differential outcomes effect, a study showed that the response sensitivity to the relatively obtained reinforcement rate in a concurrent scheduled task separated in the heterogeneous condition (positive vs. negative reinforcement) rather than in the homogeneous condition (positive vs. positive reinforcement) (Magoon et al., 2017). Pharmacological researches on rats showed that conventional antiepileptics were more effective in reducing positively reinforced behavior than in negative avoidance schedules (Roberts et al., 2008). In short, though sometimes not in the behavior level, the difference between positive and negative reinforcement exists in the underlying neural mechanisms.

### Limitations and Future Directions

Several limitations warrant future investigations. First, the effect of reinforcement type may exhibit interactions with the reward magnitude on each trial and the determinacy of the response–outcome association. We adopted a probabilistic paradigm to examine the learning effect on the probabilistic associations and the underlying neural mechanisms, whereas the effect of reinforcement type has not been compared in learning deterministic associations in electrophysiology studies. As the reward prediction error is sensitive to the reward magnitude and probability, the dopamine system may be differently involved in different reinforcement types with different reward magnitudes or probabilities. Second, in the present study, the emotions were only assessed before and after the experiment, which seems to be somewhat coarse. The results suggested that the emotional experience in negative reinforcement may be more complicated and fluctuate during and after the reinforcement process. Thus, more details about the emotional reactions during the training should be depicted under careful design in the future. Third, the combination of EEG and functional magnetic resonance imaging (fMRI) will shed light on the underlying mechanisms of different reinforcement types. According to the present results, we predict that the emotion-related system, such as the amygdala, and the hub of performance monitoring, the ACC, may show distinct activations in positive and negative reinforcement.

## Conclusion

Though the above limitations, our study provided the first investigation to directly compare the learning bias, the emotional reactions, and the FRN in four reinforcement types in separate groups, namely, positive, negative, and combined reinforcement, as well as a control condition with no money incentive. The results of the learning bias suggested that there was no behavioral difference for the distinct reinforcement types, whereas negative reinforcement, the pure avoidance motivation, will elicit distinct emotions, which may have an influence on the feedback processing indexed by the FRN. It is likely that participants experienced more negative feelings in the training process of negative reinforcement, which reduced the FRN amplitude. Then, when the experiment ended, a sense of relief made them report higher positive affect and joviality and less fatigue. Thus, the reinforcement types modulate the emotion and the associated neural mechanisms.

## Data Availability Statement

The raw data supporting the conclusions of this article will be made available by the authors, without undue reservation.

## Ethics Statement

The studies involving human participants were reviewed and approved by the Research Ethics Committee of Shaanxi Normal University. The patients/participants provided their written informed consent to participate in this study.

## Author Contributions

WR and SX conceived and designed the study. YS, MH, and YH collected the data. SX performed the data analyses and wrote the manuscript. JH, XT, and WR revised the manuscript and provided constructive discussions. All authors contributed to the article and approved the submitted version.

## Conflict of Interest

The authors declare that the research was conducted in the absence of any commercial or financial relationships that could be construed as a potential conflict of interest.
